# Supplementary material for: Recombinant IGF-1 Induces Sex-Specific Changes in Bone Composition and Remodeling in Adult Mice with Pappa2 Deficiency
Source: Int J Mol Sci. 2021 Apr 14;22(8):4048. doi: 10.3390/ijms22084048 (PMC8070906; doi:10.3390/ijms22084048)
Supplement: Supplementary file 1 [file ijms-22-04048-s001.pdf]

SUPPLEMANTARY MATERAL

Figure S1

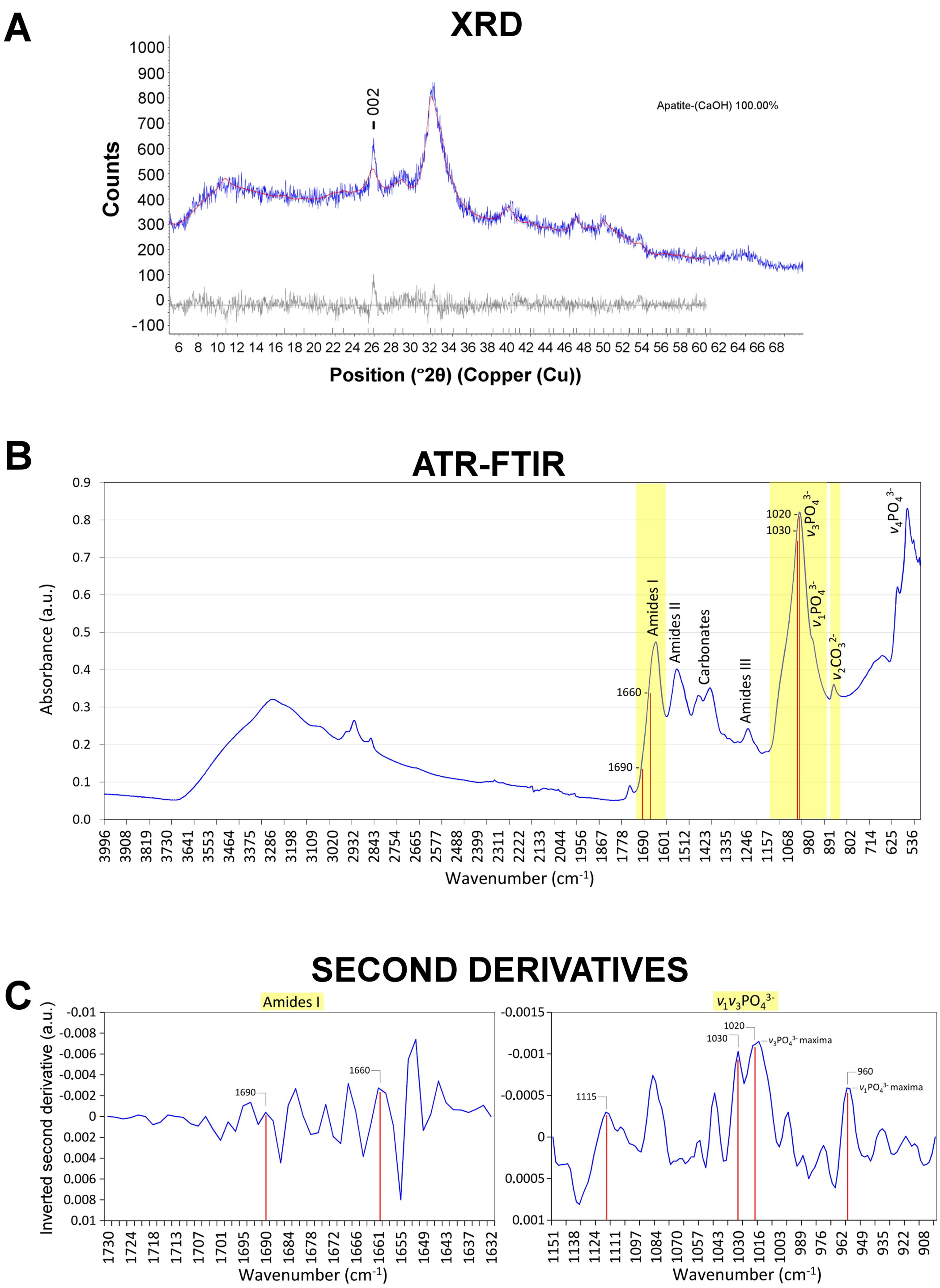

Table S1. Quantitative analysis of crystallographic properties of bone hydroxyapatite-(CaOH) in the femur of *Pappa2*$^{wt/wt}$ and *Pappa2*$^{ko/ko}$ mice (males and females) by XRD and Rietveld refinement (COD no. 9010050; Ca5 H2 O13 P3)[1].

	Male		Female	
	**wt/wt**	**ko/ko**	**wt/wt**	**ko/ko**
**R-Bragg factor (R_B)**[2]	0.985 ± 0.080	0.999 ± 0.018	0.912 ± 0.041	1.252 ± 0.313
**Cell Volume (Å^3)**	529.67 ± 0.95	529.45 ± 1.42	529.97 ± 0.36	530.53 ± 0.94
**Crystal Linear Abs. Coeff. (1/cm)**[3]	273.93 ± 0.34	273.10 ± 0.31	273.44 ± 0.18	273.16 ± 0.48
**Crystal Density (g/cm^3)**	3.153 ± 0.003	3.143 ± 0.003	3.147 ± 0.002	3.144 ± 0.005

[1] Data are represented as mean ± S.E.M. (n = 7/group). No interaction and main effects were found. See **Table 1** and **Figure S1A** for representative diffractograms.

[2] **R-Bragg factor (R_B)**: The R factor measuring the agreement between the reflection intensities calculated from a crystallographic model and those measured experimentally.

[3] **Crystal Linear Absorption Coefficient (1/cm)**: a measure of the photon beam attenuation by a material due to absorption and scattering processes.

Table S2. Quantitative analysis of spectral levels (actual absorbance) of specific ionic contents in the femur of *Pappa2*$^{wt/wt}$ and *Pappa2*$^{ko/ko}$ mice (males and females)[1].

	Male		Female	
Absorbance (a.u.)	**wt/wt**	**ko/ko**	**wt/wt**	**ko/ko**
**Phosphates ($v_1v_3PO_4^{3-}$)**	0.445 ± 0.017	0.632 ± 0.049	0.614 ± 0.048	0.621 ± 0.029
**Carbonates ($v_2CO_3^{2-}$)**	0.245 ± 0.005	0.281 ± 0.010	0.271 ± 0.007	0.263 ± 0.008
**Amides I (vC=O)**	0.425 ± 0.011	0.441 ± 0.007	0.422 ± 0.003	0.413 ± 0.009

[1] Data are represented as mean ± S.E.M. (n = 7/group). See **Figure S1B** for representative spectra.
